# Factors influencing the outcomes of colonic resections for Crohn’s colitis

**DOI:** 10.1007/s00384-024-04620-z

**Published:** 2024-05-04

**Authors:** Osman Cagin Buldukoglu

**Affiliations:** Department of Gastroenterology, Antalya Training and Research Hospital, University of Health Sciences, Varlik mah. Kazim Karabekir cad., 07100 Antalya, Turkey

I read with great interest in, and congratulate Kappenberger et al. on their study [1] entitled “Clinical outcomes and perioperative morbidity and mortality following segmental resections of the colon for Crohn’s colitis.” The study revealed that complex surgical site and complex surgical procedure increase the odds of perioperative morbidities.

Crohn’s disease is a debilitating, chronic disease that can affect the entire gastrointestinal tract. Despite the recent developments in therapeutic options with biologic and small-molecule therapies, 3 out of 4 Crohn’s disease patients require surgical interventions during their lifetime [2].

Apart from wide array of covariates included in study data by Kappenberger et al. [1], there are additional factors that can affect post-surgical outcomes, thus would alter the results of the study. Anemia, weight and nutrition status of the patient, alcohol use and smoking, as well as the surgeon’s experience have been shown to affect anastomotic leakage after colorectal surgery [3]. Urgent surgery is a risk factor for overall postoperative complications and intraabdominal septic complications in Crohn’s disease patients undergoing bowel resection [4]. The impact of magnetic resonance imaging parameters such as visceral fat, myopenia and myosteatosis on postoperative outcomes in Crohn’s disease has been studied but none of these proposed radiological findings have been found to be effective in predicting postoperative complications [5, 6].

In conclusion, the article by Kappenberger et al. contributes greatly to an important and burdensome clinical situation for both the patient and healthcare team. Future prospective studies incorporating established and proposed risk factors for surgical outcomes in Crohn’s disease patients will shed more light on the topic.
